# Overview of *Clostridium difficile* infection: implications for China

**DOI:** 10.1093/gastro/got029

**Published:** 2013-11-04

**Authors:** Xinhua Chen, J. Thomas Lamont

**Affiliations:** Division of Gastroenterology, Beth Israel Deaconess Medical Center, Harvard Medical School, Boston, MA, USA

**Keywords:** *Clostridium difficile*, *C. difficile* infection (CDI), treatment, prevention

## Abstract

The incidence and severity of *Clostridium difficile* infection (CDI) have dramatically increased in the Western world in recent years. In contrast, CDI is rarely reported in China, possibly due to under-diagnosis. This article briefly summarizes CDI incidence, management and preventive strategies. The authors intend to raise awareness of this disease among Chinese physicians and health workers, in order to minimize the medical and economic burden of a potential epidemic in the future.

## INTRODUCTION

*Clostridium difficile* is the most commonly identified cause of nosocomial diarrhea in the developed world. A steep increase in the incidence and severity of this disorder has been observed in western countries for the past several decades [1], but very limited information is available on the status of *C. difficile* infection (CDI) in China. Among an international sample of physicians from the USA, Europe and Asia, the level of awareness of this infection was inadequate [2]. Given the increasing elderly population and the well-recognized problem of over-prescription of antibiotics [3], it is important for physicians and healthcare workers in China to be aware of this global infection. This review briefly summarizes the disease incidence, current management, new treatment strategies for CDI, and its emergence in China.

## INCIDENCE AND SEVERITY

*C. difficile* is an anaerobic, gram-positive, spore-forming bacterium first isolated in 1935, but not identified until 1978 as the cause of antibiotic-associated pseudomembranous colitis [4]. Despite our growing knowledge of the epidemiology, pathogenesis and treatment of CDI during the past three decades, the infection has continued to spread globally from its initial sites in western Europe and North America to involve eastern Europe, Asia, and Australia. Furthermore, in North America and Europe, the incidence and severity of CDI and mortality rates from the disease have increased dramatically since 2000 [5]. This will probably also occur in currently low-incidence areas such as China and Japan.

Very few published reports are available in Asia in general—and China in particular—on the overall incidence of *C. difficile* infection at the national level [6]. A review of the currently available English and Chinese literature documented the presence of CDI in mainland China, but suggested that this infection was only rarely diagnosed [3]. The CDI rate in the general in-patient population of China is lower than the reported rates in western countries, according to the very limited studies conducted so far (Table 1) [7, 8]. However, CDI may be more prevalent in high-risk patients, such as those in intensive care and oncology units [3, 9]. For example, in 44 stem cell transplant patients, twelve cases (27.3%) of CDI were diagnosed [10]. Of fecal samples from 70 hospitalized patients in Hunan Province with diarrhea, who had been exposed to antibiotics, 30% were positive for *C. difficile*. Twenty-one isolates of *C. difficile* were further assigned to seven ribotypes, with the dominant types being 017 (48%), 046 (14%) and 012 (14%). However, the epidemic PCR ribotype 027 and 078 strains were not identified [11]. In contrast to the mainland, an early case of *C. difficile* belonging to the hypervirulent strain ribotype 027 was identified in Hong Kong in 2008 [12]. This triggered a survey of *C. difficile* in a defined healthcare region in Hong Kong. The investigators observed a significant increase over five years in the rate of CDI from 0.53 to 0.95 per 1000 admissions [13], a rate that is approximately one tenth the rate of CDI observed in American acute care hospitals [14]. In a 2010 study conducted in a Shanghai hospital, CDI incidence in patients exposed to antibiotics was 23.8% [15]. As the patients in these studies were all from one hospital, the reported high incidence rates may not reflect the rates in China as a whole.

**Table 1.** Reports of CDI incidence in China

City/Region	Reported CDI Incidence	Reference
Beijing	(i) 36 cases among 71 428 general patients from 1998–2001	*Wang et al. 2004*
	(ii) 12 cases from 44 patients with stem cell transplants	*Jia et al. 2008*
Shanghai	(i) 56 cases among 42 936 general patients from 2007–2008	*Huang et al. 2008*
	(ii) 20 cases among 84 patients exposed to antibiotics	*Gao et al. 2010*
Changsha	21 cases from 70 patients with diarrhea and exposed to antibiotics	*Hawkey et al. 2013*
Hong Kong	Incidence rate increase from 0.53 (period I: 2004–2008) to 0.95 (period II: 2009) per 1000 admissions	*Cheng et al. 2011*

CDI = *Clostridium difficile* infection

## RISK FACTORS

Use of antibiotics is the most important risk factor for the development of CDI, due to impairment of colonization resistance [16]. Ampicillin or amoxicillin, clindamycin, cephalosporins, and fluoroquinolones are most frequently associated with CDI [17], but almost all antibiotics have been associated with CDI. In China, despite increasingly stringent enforcements of medical guidelines, antibiotic usage is still loosely regulated in many regions of the country. Indeed many antibiotics are available without a prescription in China. Unregulated antibiotic usage may eventually increase the rate of infection in China.

Another important risk factor for *C. difficile* infection is inflammatory bowel disease (IBD), in which *C. difficile* is the most common superimposed infection [18–20], and one that is associated with worse clinical outcomes [18, 21]. IBD, originally considered a ‘western’ disease, has been reported with increasing frequency and severity in China. According to a recent report, among 10 218 mainland Chinese patients with ulcerative colitis (UC), 2506 patients were diagnosed between 1981 and 1990, whereas 7512 were diagnosed between 1991 and 2000 [22]. These figures represent a threefold increase in the number of UC cases over the two decades, perhaps related in part to increased recognition and diagnosis rather than a true increase in incidence. CDI may be difficult to distinguish from an IBD flare and thus a high level of suspicion is required. As both CDI and IBD may be under-recognized in China, it is important for physicians to be aware of the clinical features of these two emerging diseases.

Advanced age also predisposes to risk of acquisition of CDI as well as severity of infection. The elderly population continues to grow in Chinese society and, by 2026, more than 200 million Chinese citizens will be 65 or older [11, 23]. Therefore it is logical to assume that the CDI risk and severity in China will significantly increase in the future.

## TREATMENT OF CDI

Permanent cure of CDI requires restoration of the original normal colonic microflora, resulting in the elimination of *C. difficile*. Current major intervention and emerging treatment strategies are discussed below.

### Discontinuation of antibiotics

Discontinuation of antibiotics can often improve patients with mild clinical symptoms [17]. The standard initial therapy for mild CDI is to discontinue all antibiotics if possible and monitor the patient’s progress. Almost all patients are administered an oral antibiotic directed at *C. difficile*.

### Vancomycin, metronidazole and fidaxomicin

Oral administration of vancomycin and metronidazole are currently the first-line treatments for CDI. For patients with mild or moderate CDI, metronidazole is adequate. Oral vancomycin is recommended in patients with severe CDI, or those who do not respond to or cannot tolerate metronidazole, or those with multiple recurrences of CDI [24]. Vancomycin is superior to metronidazole as initial therapy for *C. difficile* infection that is considered severe as determined by the presence of high fever (>38.3°C), elevation of white blood count >15 000 cell/mm^3^, albumin < 2.5 g/dL, and age >60 years [25]. All antibiotics, including metronidazole and vancomycinimpair the fecal microbiome and its ability to resist colonization, thereby facilitating recurrent infection [1]. About 25% of patients treated with metronidazole or vancomycin will suffer a recurrence after treatment is discontinued; many of these will have multiple recurrences [26]. In May 2011, the US Food and Drug Administration approved fidaxomicin for the treatment of CDI*.* Compared with vancomycin, fidaxomicin was associated with a significantly lower rate of recurrence of CDI (25% vs 15%) [27]. However, its cost-effectiveness for the treatment of CDI remains questionable [28], as the drug is considerably more expensive than either metronidazole or vancomycin.

### Fecal microbiota transplantation

Fecal microbiota transplantation (FMT) involves the infusion of a fecal suspension from a healthy donor into the gastro-intestinal tract of a patient with colonic disease [29, 30]. With cure rates of 90–95% reported in uncontrolled trials, FMT is emerging as the best therapy for recurrent CDI [30, 31]. In the only randomized, controlled trial, FMT administrated via a nasojejunal tube resulted in resolution of *C. difficile*-associated diarrhea in 81% of patients with recurrence (vs 27 for controls receiving vancomycin) [32]. Despite the reported high cure rates, FMT has several limitations: to increase safety, screening of all FMT donors is recommended, including a careful review of their medical history, and blood and stool tests to detect any possible stool pathogens [31]. In addition, FMT is esthetically unappealing and logistically challenging. It is likely that the use of feces may eventually be replaced by a defined bacterial mixture that confers colonization resistance against *C. difficile*. Current research characterizing specific commensal bacterial species that protect against CDI may lead to such an attractive future strategy.

### Immunotherapy

Immune responses to *C. difficile* toxins are a key determinant of the outcomes of CDI [33, 34]. Kyne *et al.* reported that serum IgG antitoxins directed against toxin A were protective against CDI in hospitalized patients exposed to antibiotics [33]. Humanized monoclonal antibodies (MAbs) against *C. difficile* toxins have offered a major advance in passive immunotherapy for CDI. Intravenous infusion significantly reduced the recurrence of CDI in a large multicenter, randomized, double-blind, placebo-controlled trial [35]. The antibodies were administered in conjunction with vancomycin or metronidazole in patients with acute CDI. Compared with a 25% recurrence rate in the antibiotics alone group, only 7% of patients treated with MAbs had recurrence. Future studies will examine whether these MAbs will be cost-effective for the treatment of CDI.

### Chinese herbal medicines

Chinese herbal mixtures have been used as treatment for CDI in China [3]. For example, a herbal remedy containing *Puerariae radix*, *Scutellariae radix*, and *Rhizoma coptidis* was beneficial in treating CDI [36]. Combined herbal therapy using the ‘four miraculous drugs’ plus vancomycin was more effective when compared with vancomycin alone [37]. Garlic preparations have also been reported to improve pseudomembranous colitis [38]. Although comprehensive biological studies and randomized controlled clinical trials are currently lacking, natural products or Chinese herbal medicines as adjunctive treatment may hold promise as non-antibiotic-based alternative therapies for CDI.

## PREVENTION OF CDI

### Antibiotic stewardship

Since nearly all patients with CDI have been previously exposed to antibiotics, it is important to recognize that careful restriction of antibiotic usage to conform to clinical guidelines may help decrease hospital incidence of CDI. Studies have shown that antibiotic prescription guidelines reduce *C. difficile* infection rates by approximately 50% [39, 40]. It has been shown that good antibiotic stewardship can lead to less overall and inappropriate use of antibiotics, reductions in CDI, and less emergence of antimicrobial resistance [41]; therefore, stewardship of antibiotics, especially broad-spectrum agents, will be an important measure for CDI prevention in China, where over-prescription is widely recognized [3].

### Environmental decontamination

Use of disposable gloves and gowns, and hand washing with soaps containing chlorhexidine gluconate have all been reported to reduce the spread of *C. difficile* by healthcare workers [42]. Decontamination of rooms and equipment exposed to CDI patients is recommended, using sporicidal agents [43].

### Probiotic strategies in CDI

Probiotics are defined as live micro-organisms that confer a health benefit to the host. Since CDI is associated with disrupted fecal flora and loss of their normal barrier function, it is logical to employ probiotic strategies that modulate gut flora as prophylaxis for this infection. In a recent meta-analysis including 20 randomized trials and 3818 patients, probiotic prophylaxis reduced the incidence of CDI by 66% [44]. In a study conducted in Shanghai, the probiotic combination of *Lactobacillus acidophilus* CL1285 and *Lactobacillus casei* LBC80R were given to hospitalized patients within 36 hours of initial antibiotic administration and continued for 5 days [15]. This probiotic prophylaxis resulted in a dose-responsive and significant reduction of CDI rate (low dose: 9.4%; high dose: 1.2%) compared with placebo control (23.8%) [15]. In two other trials conducted in England, the probiotic mixture of *Lactobacillus* and *Bifidobacterium* and that of *Lactobacillus* and *Streptococcus thermophiles* both demonstrated efficacy in lowering CDI incidence without side-effects [45, 46]. However, a recent randomized, controlled trial in the UK showed no clear benefit of probiotic mixture containing *Lactobacilli* and *Bifidobacteria* in the prevention of CDI in older inpatients exposed to antibiotics in the hospital [47]. In addition to probiotic bacteria, *Saccharomyces boulardii* (Sb), a probiotic yeast, was tested in a double-blind, randomized, placebo-controlled study in patients with recurrent CDI [48]. In this study, Sb was used in combination with metronidazole or vancomycin. A majority (65%) of the control subjects (antibiotics alone) experienced recurrence, compared with only 35% of those receiving antibiotics plus Sb. However, a recent clinical trial suggested that Sb was not effective in preventing CDI in elderly hospitalized patients [49]. Lastly, the use of non-toxigenic *C. difficile* to prevent primary or recurrent CDI has been proposed as an alternative strategy [50], as asymptomatic colonization of patients with *C. difficile* (toxigenic or non-toxigenic strains) is known to be associated with decreased risk of CDI [51]. Phase II trials using non-toxigenic *C. difficile* are currently ongoing. The efficacy of this approach remains to be seen.

Given their great variety, considering probiotics as a single entity is likely to over-simplify their diverse mechanisms, functions and clinical benefits. In addition, bacteremia or fungemia attributed to probiotic administration have been reported [52, 53]. Therefore, caution should be used on immunocompromised patients—or those on immunosuppressive medication—before probiotic usage.

### Vaccine development

Vaccination would ultimately provide a cost-effective way of controlling CDI, as the pathogenesis is entirely attributable to the actions of toxin A and toxin B on the gut epithelium. Antibodies to the toxins (anti-toxins) interfere with their binding to cell surface receptors on colonic epithelial cells [54]. Based on this rationale, the first candidate vaccine against *C. difficile* was a toxoid vaccine containing formalin-inactivated, purified toxins A and B. This human vaccine was found to be safe, well-tolerated and associated with high level responses of serum antitoxin antibody [55], and was also successful in treating a small number of patients with recurrent CDI [56]. Phase II clinical trials of the toxoid vaccine for the prevention of CDI are currently ongoing. Meanwhile, a recombinant protein-based vaccine targeting the receptor binding domains of the *C. difficile* toxins adjuvanted with *S. typhimurium* flagellin induces rapid, high-level protection in a mouse model of CDI [57], therefore further pre-clinical and clinical tests are warranted. Another recombinant vaccine candidate is co-administration of a cell binding domain fragment of toxin A and the glucosyl-transferase moiety of toxin B, which induced protective immunity in hamsters [58]. As these vaccines are toxin-based, they are unlikely to affect gut colonization of *C. difficile*. To functionally target the colonization step of *C. difficile* pathogenesis, non-toxins based vaccine candidates utilizing the bacterial surface proteins or carbohydrates are also being explored [59–62].

## CONCLUSIONS

The incidence of CDI in China remains low, partly related to under-diagnosis; this has resulted in lack of recognition of CDI as a health problem. China, as the world’s most populous nation with an increasing elderly population and the well-recognized problem of antibiotics usage, must be prepared for a potential *C. difficile* epidemic. As an accurate estimate of incidence of CDI in China is not known, large-scale hospital and outpatient screening studies are needed. Routine diagnostic testing for *C. difficile* toxins should be introduced in hospitals and clinics. Better antibiotic stewardship, proper hand hygiene by heathcare workers, surveillance and prompt isolation of new cases of CDI are all recommended measures to prevent CDI. New lines of antibiotics, non-antibiotic-based approaches including FMT, immunotherapy and alternative herbal medicine—as well as vaccine development—hold promise for the treatment and prevention of CDI.

## FUNDING

Dr Xinhua Chen was supported by the Career Development Award of Crohn’s & Colitis Foundation of America.

**Conflict of interest:** none declared.
